# Decoding cooperation-oriented protective behavior amid public health crisis: How multidimensional risk communication works

**DOI:** 10.3389/fpubh.2026.1827389

**Published:** 2026-07-27

**Authors:** Yunpeng Xu, Qiaolan Liu, Jian Liang

**Affiliations:** 1 Regional Social Governance Innovation Research Center, Guangxi University, Nanning, China; 2 University of Electronic Science and Technology of China, Chengdu, China

**Keywords:** cooperation-oriented protective behavior, public health crisis, risk communication, risk perception, trust in authoritative sources

## Abstract

In the context of recurrent public health emergencies, mobilizing cooperation-oriented protective behavior is important for strengthening collective resilience and public governance. Existing research has explained many individual protective behaviors, but less is known about how the structure of the communication environment is associated with protective behaviors that also generate collective benefits. Guided by the Protective Action Decision Model (PADM) and risk communication research, this study develops a communication-structure framework encompassing information sources, communication content, message presentation formats, and communication channels. Risk perception is specified as a statistical mediating mechanism, and trust in authoritative information sources is examined as a moderating condition. Using survey data from 11 provinces in China (*N* = 1,417), the results show that the four communication dimensions are positively associated with cooperation-oriented protective behavior; risk perception shows a significant mediating pattern between risk communication and such behavior; and trust in authoritative information sources negatively moderates the association between information sources and risk perception, revealing a “high-trust attenuation” pattern. The study contributes to risk communication research by specifying PADM’s warning-information input as a structured communication environment, clarifying the appraisal role of risk perception, and showing that authoritative-source trust may operate not only as a facilitator of message acceptance but also as an information filter in multi-source public health communication.

## 1 Introduction

In today’s high-frequency crisis environment, relying solely on government capacity is insufficient to address public health crises with strong externalities and spillover effects. Such crises readily trigger “free-riding,” creating tension between individuals’ short-term rational interests and the collective’s long-term welfare, thereby generating a classic social dilemma. Therefore, promoting cooperation-oriented protective behavior has become a critical pathway for strengthening collective protective capacity and enhancing governance performance. Cooperation-oriented protective behavior refers to actions in which individuals willingly incur certain costs to achieve mutual or group gains despite conflicts between personal payoffs and collective benefits (1, 2). This cooperation is not only a prerequisite for the provision of public (or public-safety) goods, but also a source of social capital that expands interpersonal networks, fosters generalized reciprocity, and advances collaborative governance (3).

However, such cooperation does not arise spontaneously; it is shaped by how the public understands, evaluates, and responds to risk. Risk has both an objective reality and subjective construction. Under conditions of incomplete information and heterogeneous experience and culture, individuals often form judgments that diverge from “actual” risk, thereby influencing their Behavioral choices (4). Consequently, risk communication is not merely a technical process of information transmission, but a governance instrument that transforms the informational environment into cooperative action. By enhancing threat and efficacy perceptions and reducing uncertainty and strategic skepticism, risk communication suppresses free riding and increases the likelihood of cooperation (5–7). From this perspective, designing multidimensional, perceptible, and context-adaptive risk communication strategies is essential.

Although prior work has substantially advanced understanding of risk communication and protective behavior in public health emergencies (8, 9), three gaps persist. First, behaviors such as mask wearing, vaccination, quarantine, and information seeking are typically framed as individual health-protection decisions, overlooking their collective-benefit dimension: each also reduces transmission risk, protects vulnerable others, and supports public health governance. This study therefore adopts the term cooperation-oriented protective behavior to capture actions that serve both individual-protection and public-good-oriented ends simultaneously. Second, PADM applications largely treat warning information as a generic external cue, leaving the internal structure of that cue underspecified. In public health emergencies, risk information reaches individuals through a differentiated environment comprising multiple providers, varied content emphases, diverse presentation formats, and distinct media channels—structural features that a communication-theoretical account cannot afford to collapse into a single input. Third, influential health-communication and persuasion frameworks—EPPM, the RPA framework, and TNSB (10)—offer powerful accounts of threat appraisal, efficacy appraisal, and normative influence, yet none is designed to estimate information providers, communication content, presentation formats, and media channels as simultaneously modelled components of the public information environment. How these dimensions jointly shape risk appraisal and cooperation-oriented protective behavior within a unified PADM-informed framework, and whether trust in authoritative sources moderates those associations, remains unresolved.

This study makes three contributions. First, it specifies four structural dimensions of risk communication—information sources, communication content, message presentation formats, and media channels—as comparable, simultaneously estimated antecedents of risk appraisal within a PADM-informed framework. Second, it links these dimensions to cooperation-oriented protective behavior, thereby extending PADM cautiously beyond individual action to behaviors with inherent collective-benefit components. Third, it tests the moderating role of source trust, challenging the assumption that trust uniformly amplifies communication effects and proposing a high-trust attenuation pattern whereby strong trust in authoritative sources attenuates the marginal association between non-authoritative information and risk perception.

## 2 Theoretical background

### 2.1 Protective action decision model

The Protective Action Decision Model (PADM) is a classic framework for explaining how individuals and groups decide to engage in protective actions under acute risk (11). PADM posits that behavior is jointly shaped by three classes of exogenous cues: environmental cues (e.g., hazard signals, unusual noise), social cues (observing and emulating others’ actions), and risk warnings issued by authoritative information sources (68). Filtered through individual characteristics and resource endowments, these cues trigger three core perceptions—threat perception, protective efficacy perception, and stakeholder perception—which in turn inform decisions about protective action. The original PADM is intentionally comprehensive, but its full sequence is not always traversed in real-world settings. In highly time-pressured contexts, for example, messages from high-credibility authorities can precipitate rapid action, effectively bypassing parts of the cognitive pipeline (12, 13). Building on this insight, the present study retains mechanistic core of PADM while streamlining the decision pathway by collapsing the multi-stage chain into a primary route: communication strategies → risk perception → Behavioral response. This adapted specification is tailored to public health emergencies, allowing us to identify how multidimensional government risk communication is associated with variation in risk perception and, subsequently, cooperation-oriented protective behavior in social dilemma situations.

### 2.2 Risk communication as a field of communication research

Risk communication is not a single, self-contained theory, but a research domain that examines how risk information is produced, designed, transmitted, interpreted, and translated into action under uncertainty. This domain includes multiple communication-theoretical concerns, such as source credibility, message design, evidence format, narrative persuasion, channel selection, efficacy appraisal, and social influence. Accordingly, this study does not treat “risk communication theory” as an independent theory to be simply combined with PADM. Instead, it draws on risk communication research to specify the communication structure of PADM’s warning-information input in public health emergencies.

From a communication-structure perspective, public-health risk communication can be organized around four analytically separable questions: who provides the information, what content is emphasized, how the message is presented, and through which channel the information is received (14, 15). These questions correspond to four dimensions in this study: information sources, communication content, message presentation formats, and communication channels. This specification moves beyond treating warning information as a general external cue and allows the communication environment to be examined as a structured set of inputs to risk appraisal within a PADM-informed framework.

In this framework, information sources refer to the perceived actors or institutions from whom respondents obtain risk information; communication content refers to the thematic emphasis of the message; message presentation formats refer to preferences for story-based and data-based presentation; and communication channels refer to the technological or interpersonal routes through which information is accessed. These dimensions represent distinct communication behaviors: source acquisition, content attention, format preference, and channel reliance. Distinguishing them is important because source credibility, content salience, presentation format, and channel reliance may relate to risk perception and cooperation-oriented protective behavior through different communication mechanisms.

### 2.3 Integrating PADM with a communication-structure perspective

PADM explains protective action as an appraisal process in which external cues and warning information are evaluated through risk-related perceptions before individuals adopt protective responses. This appraisal logic is consistent with broader health-communication models, such as EPPM, the RPA framework, and TNSB, which also emphasize threat appraisal, efficacy beliefs, or socially embedded perceptions. However, the present study does not test these models directly. Rather, it uses PADM as the primary hazard-response framework and draws on risk communication research to specify the communication structure of PADM’s warning-information input. The key limitation of PADM for the present study is that warning information is often treated as a general external cue, with limited attention to its internal communication structure. Public-health risk communication, however, is not received as an undifferentiated message. It varies by who provides the information, what content is emphasized, how the message is presented, and through which channel it is received. Accordingly, this study conceptualizes multidimensional risk communication as the structured informational input in PADM and operationalizes it through four communication domains: information sources, communication content, message presentation formats, and communication-channel reliance.

Within this integrated framework, risk perception serves as the central appraisal mechanism. This specification follows PADM’s core logic while adapting it to public health emergencies, where protective actions often combine individual-protective and collective-benefit components (16). The study therefore examines whether risk perception shows a statistical mediating pattern in the association between the integrated multidimensional risk-communication index and cooperation-oriented protective behavior. Trust in authoritative information sources is incorporated as a boundary condition for information processing. Authoritative sources, such as government agencies, public health institutions, experts, and medical professionals, often provide salient reference points during public health emergencies. Higher trust in these sources may reduce uncertainty, but it may also narrow the range of information cues individuals use when forming risk appraisals (17). The model therefore examines whether trust in authoritative information sources attenuates the association between information sources and risk perception.

**Figure 1:**
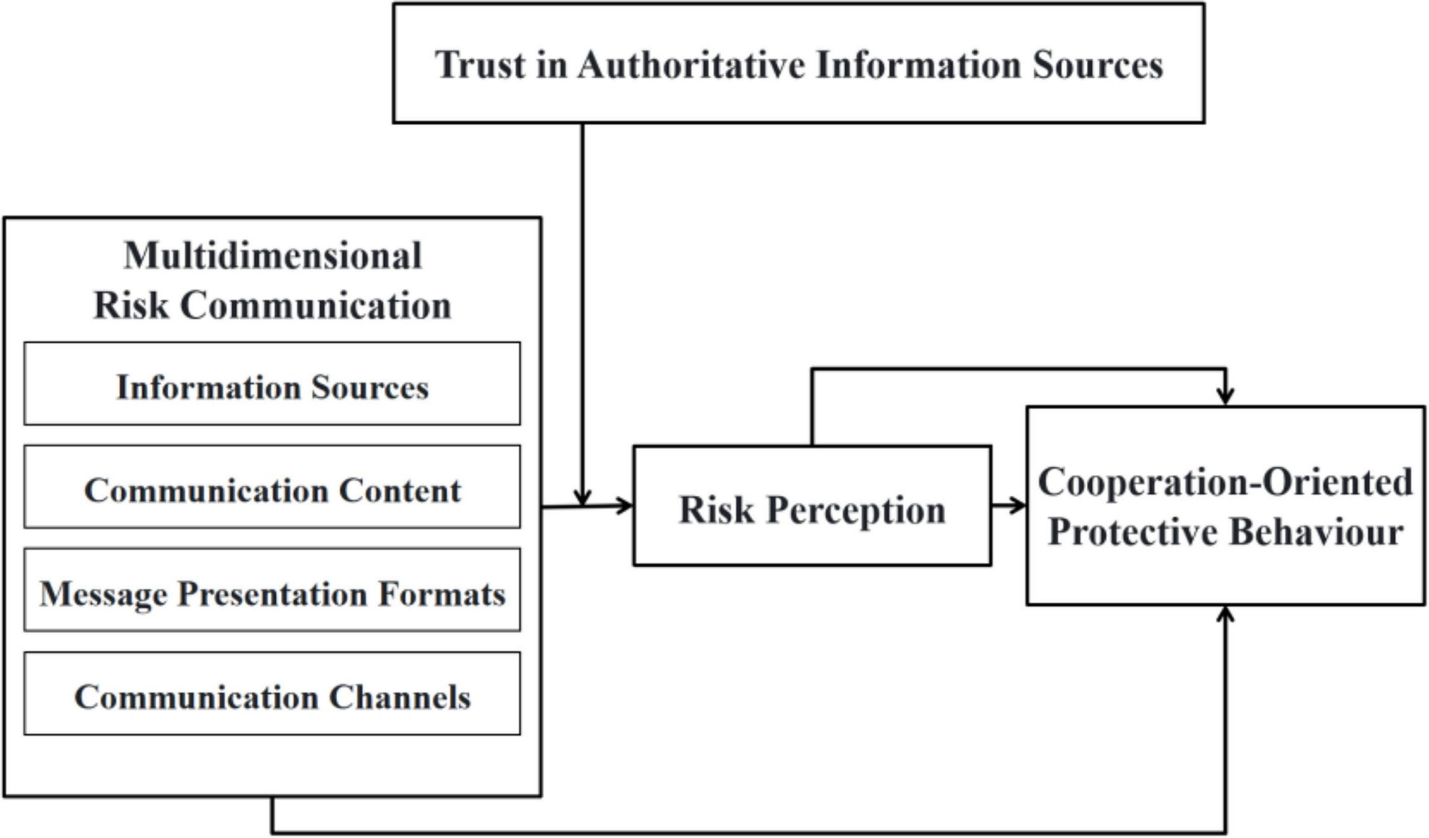
Research framework.

Taken together, this framework does not simply add communication variables to PADM. It specifies the communication structure of PADM’s warning-information input, identifies risk perception as the appraisal mechanism, and examines trust in authoritative information sources as a boundary condition. In doing so, the study links communication structure, risk appraisal, and cooperation-oriented protective behavior within a single PADM-informed model (Figure 1).

In the information source dimension, drawing on Griffin and colleagues’ typology (18) and balancing authority, reach, and personalization, we classify risk information sources in public health emergencies into three types: authoritative information sources (AIS)—including government agencies, experts, and medical personnel; media-based information sources (MIS)—include news organizations, media accounts, civil-society organizations, and social-media influencers and interpersonal information sources (IIS)—such as family members, friends, and neighbors. For communication content, following Sutton and Kuligowski and related guidance (19, 20), we distinguish three categories: epidemic information (EI), which covers origin, hazards, and casualty data; institutional response information (IRI); and preventive information (PI) on protective measures and disease control. Consistent with narrative transportation theory and prior empirical typologies (21, 22), we identify two messsage presentation formats: a story-based formats (SF) and a data-based formats (DF). Finally, for the communication channels (CC) dimension—guided by media richness theory and established survey instruments such as the China General Social Survey (CGSS)—we grouped channels into the Internet (IT), television (TV), telephone calls and SMS (TCSMS), radio broadcasts (RB), newspapers and printed materials (NPM), and face-to-face communication with public officials (F2F) (23).

## 3 Research questions

The essence of risk communication is to reshape the internal structure of a risk event through information transmission (24). Effective risk communication not only mitigates government–public misalignment, heightens perceived threat, and strengthens civic responsibility; it also activates prosocial motivation through the joint effects of information provision and perceived trust, thereby translating intentions into cooperation-oriented protective behavior, with consequential impacts on epidemic control (25). Accordingly, we propose the following hypotheses. To avoid treating each observed item as a separate theoretical claim, this study formulates one directional hypothesis for each communication domain and uses research questions to compare the relative salience of subdimensions within each domain. This structure is consistent with the communication-structure perspective developed above: the theoretical interest lies in whether different communication domains and their internal components show distinguishable associations with cooperation-oriented protective behavior.

### 3.1 Information sources and cooperation-oriented protective behavior

During public health emergencies, audiences typically process risk information by first attending to who speaks before what is said. A large body of evidence shows that source credibility significantly shapes message acceptance, risk appraisal, and subsequent protective or cooperation-oriented action (26, 27). Under conditions of high uncertainty and information overload, individuals rely more heavily on source cues to make heuristic judgments that determine whether they will engage in deeper processing and take action (28). First, authoritative information sources (AIS), such as government departments and professional agencies, possess a built-in advantage in normativity and credibility and are typically associated with higher policy compliance and collective cooperation (29). However, institutional procedures and agenda constraints may slow AIS updates in the early stages of an outbreak, prompting audiences to consult additional sources (30). Second, media-based information sources (MIS), by virtue of their broad reach and real-time diffusion, can heighten risk perception and emotional arousal, thereby increasing willingness to cooperate and adopt protective measures during crises (16, 31). Finally, interpersonal information sources (IIS) leverage “close-range persuasion” embedded in social norms and trust relations; by strengthening affective involvement and peer pressure, IIS further encourage cooperation-oriented tendencies (32). Based on this, we propose the following hypotheses and research questions:

*RQ1: During public health emergencies, which type of information source — authoritative information sources, media-based information sources, or interpersonal information sources—shows the strongest association with cooperation-oriented protective behavior?*

### 3.2 Communication content and cooperation-oriented protective behavior

In public health crises, the design of communication content is pivotal in converting risk perception into actual cooperation. The PADM posits that people act only after recognizing the threat (e.g., severity and likelihood of exposure) and believing that the recommended measures are feasible and effective (33). A meta-analysis showed that content emphasizing action guidance and efficacy information is more effective than threat-only messages in promoting preventive Behaviors (34). Likewise, Oh, Lee, and Han (16) found that social media posts containing clear prevention guidelines increase the likelihood of adopting protective or cooperation-oriented protective behavior by elevating people’s risk perception and self-efficacy. Based on this, we propose the following hypotheses and research questions:

*RQ2: During public health emergencies, which type of communication content — epidemic information, institutional response information, or preventive information — shows the strongest association with cooperation-oriented protective behavior?*

### 3.3 Messsage presentation formats and cooperation-oriented protective behavior

Narrative communication theory posits that the form in which crisis information is expressed can substantially shape emotional engagement, risk appraisal and willingness to cooperate (35, 36). Story-based formats heighten immersion and empathy through characters, plot, and affective cues, drawing audiences psychologically “into” the narrative world and, in turn, strengthening value alignment and motivation to act (37). Health stories with strong plot structures and character identification have been shown to elevate emotional resonance and self-efficacy, thereby promoting protective and cooperation-oriented protective behaviors (35, 38). In contrast, data-based formats enhance perceived credibility and scientific rigor; when rational evaluation is salient, data-centric evidence often increases persuasiveness and facilitates behavioral adoption (22). In pandemic control, clear statistics on risk and efficacy can reinforce intentions to cooperate and comply with preventive measures. Based on this, we propose the following hypotheses and research questions:

*RQ3: During public health emergencies, which message presentation format — story-based presentation or data-based presentation — shows the stronger association with cooperation-oriented protective behavior?*

### 3.4 Communication channels and cooperation-oriented protective behavior

Differences across communication channels in information richness, timeliness, and social influence pathways yield heterogeneous effects on cooperation-oriented protective behaviors. During crises, the Internet and social media can rapidly amplify official messages and mobilize public action due to their broad reach and high interactivity, while simultaneously introducing risks of information overload and distortion (39–41). By contrast, traditional media—television, radio, and newspapers—retain agenda-setting capacity and perceived authority, which tend to stabilize trust in official responses and increase adherence to collective action (42). Beyond mass media, direct-to-person channels also matter: SMS and telephone interventions are targeted, timely, and actionable, and have been shown to improve health protection and compliance (43, 44). Moreover, face-to-face communication with street-level bureaucrats strengthens understanding and trust in policies, thereby increasing cooperation in social dilemmas (45). Building on this literature and our measurement scheme, we propose the following hypotheses and research questions:

*RQ4: During public health emergencies, which communication channel — the Internet, television, radio broadcasts, newspapers and printed materials, telephone calls and SMS, or face-to-face communication with public officials — shows the strongest association with cooperation-oriented protective behavior?*

This study conceptualizes risk communication along four core dimensions—information sources, communication channels, communication content, and message presentation formats—and advances specific hypotheses for each dimension. Building on these dimensions, we applied the entropy and CRITIC methods to derive the combined weights and construct an integrated risk communication index. Based on this, we propose the following hypothesis:

*RQ5: Is the integrated multidimensional risk-communication index, combining information sources, communication content, message presentation formats, and communication channels, positively associated with cooperation-oriented protective behavior?*

### 3.5 Mediating role of risk perception

Distinct risk communication strategies influence risk perception through multiple pathways, producing differentiated psychological and Behavioral responses (66). Variations in the authority, expertise, and social distance of information sources condition judgments of credibility and, in turn, Behavioral intentions (46). The completeness, reliability, and action orientation of communication content directly affect decision-making (33). Different messsage presentation formats elicit distinct psychological effects: vivid, story-based formats heighten emotional engagement and empathy, raising risk awareness and willingness to act (36), whereas structured, data-based presentations strengthen analytical processing and perceived efficacy (22). As carriers of information flow, communication channels shape accessibility, interactivity, and perceived social endorsement, which in turn affect the depth of exposure and processing, thus exerting a strong influence on risk perception (42). Perceived susceptibility, severity, and coping efficacy are core psychological predictors of protective Behaviors (47). In acute public health emergencies, elevating risk perception promotes self-protection and cooperation-oriented protective behavior in social dilemmas (48). Accordingly, we posit the following hypothesis:

*RQ6: Does risk perception show a statistical mediating pattern in the associations between multidimensional risk-communication domains and cooperation-oriented protective behavior?*

### 3.6 Moderating role of trust in authoritative information sources

Public trust in information sources typically hinges on their perceived authority, expertise, transparency, and reliability (49). Two patterns of trust variations were observed. First, cross-source differences: in digital environments, trust in governmental agencies and medical experts generally exceeds that in commercial platforms or social media (50). Although medical professionals are often viewed as the most credible health information providers, people frequently consult the Internet and other mass media first when making health decisions (70). When trust in authoritative sources is lacking, uncertainty and anxiety increase, amplifying the risk perception (51). Second, within-source heterogeneity: individual characteristics, information literacy, and prior experience lead to divergent levels of trust in the same source across groups, yielding different risk perceptions and Behavioral responses (52). Conceptually, trust functions as a heuristic that reduces processing complexity and cognitive costs (65). In public health crises, high-trust individuals are more receptive to official risk communication and recommended protective measures, whereas low trust can heighten anxiety and perceived risk (53). Building on this logic, we posit the following hypothesis:

*RQ7: Does trust in authoritative information sources moderate the relationship between information sources and risk perception?*

## 4 Materials and method

### 4.1 Data and sample

We conducted an online survey using Credamo from October to December 2023. All respondents provided informed consent and voluntarily participated. In total, 1,491 questionnaires were collected from 11 provincial regions in China. After excluding invalid entries, 1,417 valid responses remained, yielding a valid-questionnaire rate of 95.04%. Women comprised 51.7% of the sample population. The age distribution was concentrated among younger and middle-aged adults (18–26:32.1%; 27–39:42.6%; 40–60:19.0%). Of these, 51.5% reported non-agricultural household registration, and 80.4% self-rated their health as good. The measurement items were informed by prior research and adapted to the Chinese public health context. Because the study combines multi-item reflective constructs with conceptually distinct single-item communication indicators and weighted composite indices, the measures are not treated as identical psychometric scales.

### 4.2 Measures

The survey measured individual-level perceptions, self-reported communication experiences, and self-reported cooperation-oriented protective behavior in epidemic-related public health emergencies. The communication measures were designed to capture recipient-side engagement with risk information rather than the objective properties of specific messages or channels. This approach is consistent with the PADM-informed focus on warning-information input and protective-action appraisal (33). Accordingly, the conceptual communication domains and their empirical operationalizations are distinguished in this study. Information sources were operationalized as information-source acquisition; communication content was operationalized as communication-content attention; message presentation formats were operationalized as message-format preference; and communication channels were operationalized as communication-channel reliance.

The item wording determines the interpretation of each communication measure. Source items asked whether respondents obtained information from particular source types. These items therefore capture reported acquisition rather than source preference, source credibility, or source trust. Content items asked whether respondents focused on particular types of epidemic-related information. These items capture reported attention rather than understanding, objective knowledge, recall accuracy, or information acceptance. This distinction is important because attention and comprehension are separate stages of mediated information processing (54). Format items asked whether respondents preferred story-based or graphical-data presentations. These items capture format preference rather than actual exposure to specific messages or persuasive effects of message format. Channel items asked whether respondents relied on particular routes of access. These items capture channel reliance rather than objectively recorded media use, exposure duration, or channel effectiveness.

This operationalization is consistent with the PADM-informed focus on warning-information input, but it also sets clear boundaries for interpretation. The four communication domains are conceptually related because they describe how respondents encountered and oriented to risk information, yet they are not parallel reflective indicators of a single latent communication construct. For this reason, Cronbach’s alpha is not reported for IS, CC, MPF, or CH; these domains are composed of conceptually distinct single-item indicators. The means, standard deviations, reliability coefficients, and measurement items for each variable are presented in Tables 1 and 2, respectively.

**Table 1:** Measurement items of the variable.

Variables	Dimension	Item	Reference
Information source (IS)	AIS	Do you get information about public health emergencies from government departments or experts?	Griffin et al. (18)
	MIS	Do you obtain information related to public health emergencies from mass media such as news organizations, media accounts or various social media influencers?	
	IIS	Do you get information related to public health emergencies from your family and friends?	
Communication content (CC)	EI	Do you focus on the source of the epidemic, the hazards, the number of deaths and injuries, etc.?	Sutton and Kuligowski (19) and Seeger et al. (20)
	IRI	Do you focus on information about how relevant government departments are preventing and handling the epidemic?	
	PI	Do you focus on knowledge and information about the prevention and treatment of the epidemic?	
Messsage presentation formats (MPF)	SF	Do you prefer to learn about the epidemic through specific people and stories?	Zebregs et al. (22)
	DF	Do you prefer graphical data to learn information about the epidemic?	
Communication channels (CH)	IT	Do you rely on the Internet to search for and receive information related to the epidemic?	Chan et al. (23)
	TCSMS	Do you rely on telephone calls or SMS to receive epidemic-related information?	
	TV	Do you rely on television to search for and watch epidemic-related information?	
	RB	Do you rely on radio to listen to information about the epidemic?	
	NPM	Do you rely on newspapers or printed materials to search and read information about the epidemic?	
	F2F	Do you rely on face-to-face communication from government officials to get information about the epidemic?	
Trust in authoritative information source (TIAIS)		Do you feel that the information released by government and healthcare professionals is professional and accurate?	Siegrist and Zingg (58)
		Do you feel that the information released by government and healthcare professionals is transparent and credible?	
		Do you feel that the information released by government and healthcare professionals is complete and unbiased?	
Risk perception (RP)		Do you believe the epidemic poses a serious threat to you and your family’s physical and mental health?	Slovic (67)
		Do you think it is likely that you and your family will get infected or be infected?	
		Do you think it would have a significant negative impact on your and your family’s life and work?	
		Do you feel worry about the epidemic?	
		Do you feel anxious about the epidemic?	
		Do you feel fearful about the epidemic?	
Cooperation-oriented protective behavior (COPB)		Do you take the initiative to undergo virus testing??	Oh et al. (16) and Vartti et al. (69)
		Do you scan the health code and venue code?	
		Do you comply with the call to stay at home for quarantine?	
		Do you cooperate with government requirements to quarantine at a centralized facility?	
		Do you receive a vaccination?	
		Do you suspend work or production activities (including employment, manufacturing, animal husbandry, or farming) as required by the government?	

The survey introduction asked respondents to answer the questions with reference to epidemic-related public health emergencies. It did not restrict respondents to a single named outbreak. Therefore, the responses should be interpreted as retrospective individual reports of epidemic-related public health emergencies rather than event-specific behavioral records.

**Table 2:** Properties of the main variables.

Variables	Cronbach’s *α*	Mean	SD	Dimension
IS	—	4.08	1.03	AIS
		4.18	0.948	MMIS
		3.7	1.056	IIS
CC	—	4.3	0.826	EI
		4.31	0.809	IRI
		4.41	0.76	PI
MPF	—	3.4	1.016	SF
		4.04	0.933	DF
CH	—	4.27	0.869	IT
		3.12	1.228	TCSMS
		3.42	1.153	TV
		3.05	1.306	RB
		2.84	1.301	NPM
		3.41	1.209	F2F
TIAIS	0.901	3.75	0.977	
RP	0.833	3.67	0.8	
COPB	0.918	4.37	0.662	

Cronbach’s α is reported only for reflective multi-item scales. IS, CC, MPF, and CH are composite domains formed from conceptually distinct single-item indicators; therefore, internal consistency is not applicable. “—” indicates not applicable.

Cooperation-oriented protective behavior was measured using six public-health-related behaviors, including virus testing, health-code scanning, home quarantine, centralized quarantine, vaccination, and temporary suspension of work or production when required by the government. We do not treat these behaviors as pure social-dilemma cooperation. Rather, they are public-health protective behaviors with cooperative and public-good-oriented components. They may provide some individual protection, but they also typically involve personal costs, inconvenience, or restrictions on individual freedom, while producing collective benefits by reducing infection transmission and supporting public health governance. Thus, the measurement focuses on the cooperation-oriented dimension of these behaviors rather than claiming that they are exclusively altruistic or purely collective-action behaviors.

Trust in authoritative information sources was operationalized as respondents’ evaluation of information released by government and health-professional sources in terms of professionalism, accuracy, transparency, credibility, completeness, and impartiality. This construct therefore captures trust in authoritative health-risk information rather than general political trust, generalized institutional trust, or trust in all possible information sources.

Risk perception was operationalized as a combination of cognitive and affective appraisals of epidemic risk, including perceived threat, perceived likelihood of infection, perceived life and work impact, worry, anxiety, and fear. The measure captures subjective risk appraisal rather than objective epidemiological risk.

### 4.3 Method

We applied Harman’s single-factor test to assess the potential common method bias. An unrotated principal component analysis extracted 14 factors with eigenvalues greater than 1; the first factor explained 18.59% of the variance, which is substantially below the conventional 40% threshold, indicating that common method bias is unlikely to threaten the results. Recognizing that respondents may assign different salience to the four dimensions of risk communication—information sources, communication channels, communication content, and messsage presentation formats—we combined the entropy CRITIC method to derive composite weights and construct integrated measurement indices for each dimension. The weighted indices were used to construct composite scores for the four communication domains. The detailed weights and index values are presented in Table 3. Risk perception, trust in authoritative information sources, and cooperation-oriented protective behavior were computed as weighted averages of their respective item scores, with higher values indicating stronger levels of the underlying construct. Unless otherwise specified, all items were measured on five-point Likert scales, with higher scores reflecting greater endorsement or importance attributed to the corresponding dimension.

**Table 3:** Risk communication dimensional profiles.

Variable	Dimension	EM	CRITIC	CW	Dimension	EM	CRITIC	CW
RC	IS	22.20%	25.99%	24.095%	AIS	33.05%	35.94%	34.50%
					MIS	25.69%	31.35%	28.52%
					IIS	41.26%	32.71%	36.99%
	CC	15.11%	15.95%	15.53%	EI	36.79%	33.59%	35.19%
					IRI	34.51%	32.53%	33.52%
					PI	28.7%	33.88%	31.29%
	MPF	23.20%	24.49%	23.845%	SF	58.41%	50%	54.21%
					DF	41.59%	50%	45.80%
	CH	39.49	35.57%	36.53%	TCSMS	18.36%	14.75%	16.56%
					TV	12.76%	14.97%	13.87%
					RB	23.19%	13.89%	18.54%
					PM	27.1%	13.84%	20.47%
					F2F	14.61%	15.55%	15.08%
					ITS	3.98%	27%	15.49%

SPSS 26.0 was used to conduct a multiple linear regression analysis of the effect of risk communication on cooperation-oriented protective behavior. Stepwise regression analyses were conducted to test the mediating effect of risk perception. The PROCESS macro (Model 1) was used to test the moderating effect of trust in authoritative information sources (5,000 bootstrap samples, 95% confidence interval).

## 5 Results

### 5.1 Relationship between risk communication and cooperation-oriented protective behavior

As shown in Table 4 (Models 1–2), risk communication was positively associated with cooperation-oriented protective behavior (*β* = 0.374, *p* < 0.001), thereby addressing RQ5. Among the control variables, both gender and self-rated health status showed stable and significant relationships with cooperation-oriented protective behavior. Specifically, during public health crises, women are more inclined than men to engage in cooperation-oriented actions, and individuals reporting better health exhibit a stronger willingness to cooperate.

**Table 4:** Statistical associations of risk communication and information sources with cooperation-oriented protective behavior.

Variable	Cooperation-oriented protective behavior					
	Model 1	Model 2	Model 3	Model 4	Model 5	Model 6
Constant	4.106^***^	2.767^***^	3.488^***^	3.328^***^	3.73^***^	3.098^***^
Control variable	Yes	Yes	Yes	Yes	Yes	Yes
RC		0.374^***^				
AIS			0.167^***^			0.117^***^
MIS				0.191^***^		0.143^***^
IIS					0.101^***^	−0.002
*R* ^2^	0.049	0.158	0.115	0.122	0.074	0.149
*F*	10.373^***^	32.993^***^	22.86^***^	24.419^***^	14.02^***^	24.659^***^

* = *p* < 0.05; ** = *p* < 0.01; *** = *p* < 0.001.

To improve readability, the regression tables (Tables 4–9) report only the key independent variables, mediators, moderators, and interaction terms. Control variables, including gender, age, occupation, hukou, education, health status, and political status, were included in all models but omitted from the main tables for brevity.

### 5.2 Statistical associations of information sources with cooperation-oriented protective behavior

As shown in Table 4 (Models 3–5), authoritative information sources (AIS) (*β* = 0.167, *p* < 0.001), media-based information sources (MIS) (*β* = 0.191, *p* < 0.001), and interpersonal information sources (IIS) (*β* = 0.101, *p* < 0.001) was each positively associated with cooperation-oriented protective behavior. MIS shows the strongest association among the three source types, thereby addressing RQ1. To account for the diversified risk-information environment during public health emergencies, we included all three source types in a single regression. The results in Table 4 (Model 6) show that MIS and AIS remain positively associated with cooperation, whereas the association of IIS becomes non-significant.

### 5.3 Statistical associations of communication content with cooperation-oriented protective behavior

The results in Table 5 indicate that attention to epidemic information (EI) (*β* = 0.288, *p* < 0.001), institutional response information (IRI) (*β* = 0.296, *p* < 0.001), and preventive information (PI) (*β* = 0.350, *p* < 0.001) was positively associated with cooperation-oriented protective behavior. In the separate models, the coefficient magnitudes were numerically ordered as PI > IRI > EI, thereby addressing RQ2. Because these content types frequently co-occur in public health emergencies—for example, government-issued response plans paired with guidance on citizen protective actions—we jointly estimated a model that included all three content categories. In the joint model, EI, IRI, and PI remained significantly and positively associated with cooperation-oriented protective behavior.

**Table 5:** Statistical associations of communication content with cooperation-oriented protective behavior.

Variable	Cooperation-oriented protective behavior				
	Model 1	Model 2	Model 3	Model 4	Model 5
Constant	4.106^***^	3.107^***^	3.032^***^	2.766^***^	2.569^***^
Control Variable	Yes	Yes	Yes	Yes	Yes
EI		0.288^***^			0.107^***^
IRI			0.296^***^		0.099^***^
PI				0.35^***^	0.211^***^
*R* ^2^	0.049	0.176	0.175	0.205	0.233
*F*	10.373^***^	37.478^***^	37.462^***^	45.522^***^	42.615^***^

* = *p* < 0.05; ** = *p* < 0.01; *** = *p* < 0.001.

### 5.4 Statistical associations of message presentation formats with cooperation-oriented protective behavior

As reported in Table 6, preference for both the story-based formats (SF) (*β* = 0.165, *p* < 0.001) and the data-based formats (DF) (*β* = 0.168, *p* < 0.001) was positively associated with cooperation-oriented protective behavior. In the separate models, the standardized coefficient for DF was numerically slightly larger than that for SF. When the two message presentation formats were entered simultaneously into the same regression model, both associations remained statistically significant, and the standardized coefficient for SF was numerically slightly larger than that for DF. Thus, the relative ordering of the two formats varied across model specifications, thereby addressing RQ3.

**Table 6:** Statistical associations of message presentation formats with cooperation-oriented protective behavior.

Variable	Cooperation-oriented protective behavior			
	Model 1	Model 2	Model3	Model4
Constant	4.106^***^	3.425^***^	3.556^***^	3.185^***^
Control variable	Yes	Yes	Yes	Yes
SF		0.165^***^		0.125^***^
DF			0.168^***^	0.123^***^
*R* ^2^	0.049	0.108	0.104	0.134
*F*	10.373^***^	21.266^***^	20.46^***^	24.214^***^

* = *p* < 0.05; ** = *p* < 0.01; *** = *p* < 0.001.

### 5.5 Statistical associations of communication channels with cooperation-oriented protective behavior

As shown in Table 7, reliance on the Internet (IT) (*β* = 0.235, *p* < 0.001), telephone calls and SMS (TCSMS) (*β* = 0.061, *p* < 0.001), television (TV) (*β* = 0.100, *p* < 0.001), radio broadcasts (RB) (*β* = 0.070, *p* < 0.001), newspapers and printed materials (NPM) (*β* = 0.030, *p* < 0.05), and face-to-face communication with public officials (F2F) (*β* = 0.085, *p* < 0.001) were each positively associated with cooperation-oriented protective behavior. In terms of individual effect magnitudes, the order was IT > TV > F2F > RB > TCSMS > NPM, thereby addressing R4. When all communication channels were entered jointly into a single regression model, IT, TV, RB, and F2F remained significant positive associated with cooperation, whereas the association for TCSMS became non-significant. NPM showed a significant negative association in the joint model.

**Table 7:** Statistical associations of communication channels with cooperation-oriented protective behavior.

Variable	Cooperation-oriented protective behavior							
	Model 1	Model 2	Model 3	Model 4	Model 5	Model 6	Model 7	Model 8
Constant	4.106^***^	3.201^***^	3.889^***^	3.734^***^	3.833^***^	3.999^***^	3.729^***^	2.94^***^
Control variable	Yes	Yes	Yes	Yes	Yes	Yes	Yes	Yes
IT		0.235^***^						0.216^***^
TCSMS			0.061^***^					−0.016
TV				0.1^***^				0.053^**^
RB					0.07^***^			0.07^**^
NPM						0.03^*^		−0.088^***^
F2F							0.085^***^	0.055^**^
*R* ^2^	0.049	0.141	0.061	0.077	0.065	0.052	0.071	0.166
*F*	10.373^***^	28.989^***^	11.346^***^	14.61^***^	12.169^***^	9.638^***^	13.503^***^	21.574^***^

* = *p* < 0.05; ** = *p* < 0.01; *** = *p* < 0.001.

### 5.6 Mediating role of risk perception

The stepwise regressions in Table 8 show that risk communication is positively associated with risk perception (*β* = 0.477, *p* < 0.001), and that risk perception is, in turn, positively associated with cooperation-oriented protective behavior (*β* = 0.149, *p* < 0.001). These results indicate a significant positive mediating effect of risk perception on the pathway from risk communication to cooperation, thereby addressing RQ6. Disaggregated analyses further reveal that information sources (*β* = 0.249, *p* < 0.001, communication content (*β* = 0.546, *p* < 0.001), messsage presentation formats (*β* = 0.272, *p* < 0.001), and communication channels (*β* = 0.267, *p* < 0.001) were each positively associated with risk perception, which in turn promotes cooperation-oriented protective behavior.

**Table 8:** Statistical mediating pattern of risk perception in the relationship between risk communication and cooperation-oriented protective behavior.

Variable	Risk perception					Cooperation-oriented brotective behavior				
	Model 1	Model 2	Model 3	Model 4	Model 5	Model 6	Model 7	Model 8	Model 9	Model 10
Constant	2.451^***^	3.211^***^	2.835^***^	3.145^***^	3.138^***^	2.401^***^	2.598^***^	2.270^***^	2.632^***^	2.934^***^
Control variable	Yes	Yes	Yes	Yes	Yes	Yes	Yes	Yes	Yes	Yes
RC	0.477^***^					0.303^***^				
RP						0.149^***^	0.180^***^	0.132^***^	0.175^***^	0.201^***^
IS		0.249^***^					0.199^***^			
CC			0.546^***^					0.005^***^		
MPF				0.272^***^					0.200^***^	
CH					0.267^***^					0.088^***^
*R* ^2^	0.135	0.072	0.111	0.084	0.094	0.186	0.169	0.243	0.175	0.135
*F*	27.565^***^	13.728^***^	21.886^***^	16.191^***^	18.181^***^	35.702^***^	33.09^***^	50.228^***^	33.106^***^	24.382^***^

* = *p* < 0.05; ** = *p* < 0.01; *** = *p* < 0.001.

**Table 9:** Statistical moderating pattern of trust in authoritative information sources on the relationship between information sources and risk perception.

Variable	Risk Perception			
	Model 1	Model 2	Model 3	Model 4
Constant	2.369^***^	3.260^***^	2.488^***^	2.837^***^
Control variable	Yes	Yes	Yes	Yes
IS	0.462^***^			
AIS		0.220^***^		
MIS			0.378^***^	
IIS				0.322^***^
TIAIS	0.238^**^	0.134^*^	0.283^**^	0.220^**^
IS* TIAIS	−0.060^**^			
AIS* TIAIS		−0.029		
MIS* TIAIS			−0.058^**^	
IIS*TIAIS				−0.048^**^
*R* ^2^	0.077	0.081	0.065	0.059
*F*	11.756^***^	12.444^***^	9.809^***^	8.766^***^

* = *p* < 0.05; ** = *p* < 0.01; *** = *p* < 0.001.

### 5.7 Moderating role of trust in authoritative information sources

Moderation analysis indicated a significant negative interaction between trust in authoritative information sources and information-source acquisition in relation to risk perception (*β* = −0.06, *p* < 0.01). In other words, when public trust in authoritative sources is high, the positive association of information sources on risk perception was weaker. This finding suggests that trust does not invariably strengthening the association of information; rather, the role of trust is context-dependent and structurally complex. Further dimension-specific tests revealed that the interaction of trust in authoritative information sources on the relationship between authoritative information sources and risk perception was not significant (*β* = −0.029, *p* > 0.1). However, significant negative interactions were observed for both media-based (*β* = −0.058, *p* < 0.01) and interpersonal information sources (*β* = −0.048, *p* < 0.01), thereby addressing RQ7.

## 6 Discussion

### 6.1 Communication-domain associations with cooperation-oriented protective behavior

This study found that information sources show a significant positive association on cooperation-oriented protective behavior, with standardized coefficients following a clear gradient: media-based information sources > authoritative information sources > interpersonal information sources. With their extensive reach, high timeliness, and strong agenda-setting capacity, media-based information sources showed the largest association of collective cooperation during public health emergencies (42). The results suggest that in today’s digitized and mobile media ecology, the public tends to rely more on mass-oriented channels, such as social media and online news, to obtain rapid updates and adjust cooperation-oriented protective behavior accordingly when confronting public risks (55). Although authoritative information sources remain important, their relative influence has weakened, indicating that institutional trust is no longer the sole driver of cooperation-oriented action. This finding is consistent with the “polycentric information competition” perspective in risk-communication research: when government or official messages coexist with a flood of real-time content on social platforms, audiences draw on multiple sources and engage in self-judgment, thereby reducing their dependence on any single authority (16).

At the interpersonal level, the independent effect becomes non-significant once multiple sources are modeled together, suggesting that its association with cooperation-oriented protective behavior may overlap with those of authoritative and media-based information sources. This trend reflects the deep embedding of mobile social media within risk communication networks and signals the challenges faced by community-based information channels during large-scale emergencies (56). Overall, these findings highlight the differentiated impact of information sources on cooperation-oriented protective behavior in the digital era and provide practical guidance for governmental and public agencies: strengthen collaboration and content alignment with mass media platforms while enhancing the interactivity and shareability of authoritative messages to preserve their central role in risk governance.

For communication content, preventive information showed the strongest association with cooperation-oriented protective behavior, followed by institutional response information and epidemic information. This pattern is not surprising, because the preventive-information item asks respondents whether they focus on knowledge about prevention and treatment, which is conceptually closer to several protective behaviors than the other two content categories. We therefore interpret this result cautiously. Its value lies less in showing that preventive information “works” and more in illustrating the relative salience of action-oriented content within the communication-content domain (57). The strong positive association between institutional response information and cooperation-oriented protective behaviors is consistent with the relevance of institutional authority during crises and aligns with research showing that institutional trust enhances public compliance and cooperation (58). Although basic epidemic information shows a comparatively smaller association, it provides an essential situational framework for public risk appraisal and subsequent action. Overall, the motivational hierarchy of information content can be summarized as follows: preventive directives supply operational guidance, institutional response information confers legitimacy, and epidemic information establishes the cognitive context of risk. Accordingly, public health communication should evolve from simple case reporting to a hybrid model of authoritative endorsement plus actionable directives to maximize the public’s willingness to cooperate.

Both story-based and data-based messsage presentation formats were positively associated with cooperation-oriented protective behavior. When examined separately, the standardized coefficient for DF was numerically slightly larger than that for SF., suggesting that rational, evidence-oriented communication strengthens the cognitive foundation for cooperation, consistent with prior findings that factual evidence enhances risk perception and protective intentions (59). However, when the two messsage presentation formats were entered simultaneously into the regression model, the standardized coefficient for SF exceeded that for DF, indicating that when both types of information coexist, emotion-driven storytelling elicits stronger public resonance and collective action. Narrative communication enhances memorability and persuasiveness through emotional engagement and character identification (36), aligning with the affective pathway of dual-process theory: emotional involvement heightens risk perception and activates cooperation-oriented motivation (60). These findings suggest that in public health crisis communication, data presentation alone can establish a rational baseline, but integrating storytelling more effectively mobilizes collective action. Therefore, governmental and public agencies may consider balancing empirical evidence with narrative content, employing a “data-supported plus contextualized storytelling” approach.

Multiple communication channels were positively associated with cooperation-oriented protective behavior, with the standardized coefficients numerically ordered as follows: IT > TV > F2F > RB > TCSMS > NPM. When the media were entered jointly into a single regression model, all channels except TCSMS remained significant positive predictors. This pattern underscores the centrality of digital media in risk communication, as the Internet functions as the strongest driver during public crises, owing to instantaneous dissemination, interactive feedback, and diffusion through social networks (61). Television retains substantial influence, indicating that legacy mainstream media continue to provide broad coverage for framing risks and conveying authoritative information (39). Although F2F communication is constrained by reach, it plays an irreplaceable role in strengthening public trust and willingness to comply (57). The stable effects of NPM and RB point to the enduring impact of traditional outlets among specific populations, especially older adults and communities with limited Internet access (62). In contrast, the attenuated effect of TCSMS in a multichannel environment suggests declining marginal utility; it is better suited as a supplementary or emergency prompt. Taken together, these results reveal a media hierarchy in the digital era: the Internet at the core, broadcast television providing authoritative endorsement, and face-to-face communication consolidating trust. For public health messaging, this implies prioritizing real-time interaction on online platforms while maintaining coordination with traditional media and investing in offline trust building, thereby achieving population-wide, multi-channel coverage of risk information.

### 6.2 Risk perception and trust as appraisal and boundary mechanisms

Risk perception showed a statistical mediating pattern in the association between multidimensional risk communication and cooperation-oriented protective behavior. Beyond mere information transfer, risk communication is a key driver of public cognition and willingness to cooperate (63). When risks are accurately perceived, individuals are more likely to engage in collective protection and coordinated actions (53). Notably, the effects of information sources and messsage presentation formats were particularly salient, indicating that authoritative and emotionally engaging messages are more effective in shaping risk cognition. These findings suggest that public health communication should prioritize strengthening risk perception as a core objective and align the communication content, media, and messsage presentation formats accordingly. For example, coupling data-based evidence with story-based expression and synchronizing dissemination across multiple channels can maximize the public’s subjective risk appraisal and willingness to cooperate (57).

Trust in authoritative information sources showed a negative moderation pattern for the association between information sources and risk perception. This pattern was concentrated in media-based information and interpersonal information sources, whereas the interaction involving authoritative information sources was not significant. This pattern suggests that when the public places high trust in official sources, they prioritize authoritative information and become less sensitive to and less likely to adopt content from non-authoritative sources, thereby weakening the influence of mass media and interpersonal communication on risk cognition (64). These findings refine traditional risk communication theory by challenging the assumption that trust necessarily enhances communicative efficacy. Trust is not purely facilitative; it can act as an interfering variable in alternative information pathways. Under conditions of limited cognitive resources and path dependence, individuals engage in a cognitive filtering process that produces an information-focus effect: high trust in authority reduces the reception and deep processing of diverse information (58). For public agencies, the implication is clear: while reinforcing authoritative messaging is important, fostering a balanced, multi-source information environment is equally critical. Encouraging diverse channels and avoiding overconcentration of trust help maintain the breadth and resilience of public risk perception.

### 6.3 Theoretical and practical implications

Theoretically, this study contributes to risk communication research in three ways. First, it specifies the communication structure of PADM’s warning-information input. Rather than treating warning information as an undifferentiated external cue, the study distinguishes information sources, content emphases, message presentation formats, and channels. This specification makes it possible to compare the relative salience of different communication dimensions within a single appraisal model and responds to the need for closer attention to message design and format in public health risk communication.

Second, the study clarifies the behavioral scope of PADM in public health emergencies. The outcome is not framed as pure social-dilemma cooperation. Instead, it is conceptualized as cooperation-oriented protective behavior, a form of public-health protective action that combines individual-protective and collective-benefit components. This framing avoids overstating the novelty of the behavior itself and instead focuses the contribution on how communication structure and risk appraisal are linked to such behavior.

Third, the moderation results refine the role of trust in multi-source risk communication. Trust in authoritative information sources was not simply a universal enhancer of communication effects. Its negative moderation of the association between non-authoritative information sources and risk perception suggests that trust may function as an information filter: when authoritative sources are strongly trusted, individuals may rely less on mass media and interpersonal sources in forming risk appraisals. This high-trust attenuation pattern adds a boundary-condition perspective to research on source credibility and trust in crisis communication.

Practically, these findings suggest that public health agencies should build a coordinated multi-source communication system rather than rely only on official announcements. Authoritative sources remain essential, but mass media, digital platforms, and community networks may also help broaden communication reach and facilitate timely interaction. Communication content should prioritize actionable preventive guidance, given that preventive information showed the largest association with cooperation-oriented protective behavior among the content categories examined. Crisis messages should therefore move beyond reporting case numbers or institutional responses and provide clear guidance on testing, quarantine, medical help-seeking, and protection of vulnerable groups.

Risk communicators should also combine data-based and story-based formats. Data may support credibility and rational evaluation, whereas stories may foster emotional engagement and identification. A potentially useful strategy is to use data to support factual clarity and stories to make risks and protective actions personally meaningful. Finally, communication channels should be tailored to audience habits: the Internet for rapid updates and interaction, television for authoritative framing, face-to-face communication for direct community engagement, and SMS or telephone messages for targeted emergency reminders. Public health communication should maintain authoritative consistency while preserving a pluralistic information ecology.

## 7 Conclusion

Grounded in the Protective Action Decision Model (PADM) and risk communication research, this study examined how four structural dimensions of public-health risk communication —information sources, communication content, message presentation formats, and communication channels—are associated with cooperation-oriented protective behavior during public health emergencies. The results show that these communication dimensions are positively associated with cooperation-oriented protective behavior, with risk perception showing a cross-sectional indirect-association pattern. These findings support a PADM-informed communication-structure perspective by showing that warning information can be unpacked into distinct communication domains rather than treated as a single undifferentiated cue.

The study also identifies a high-trust attenuation pattern: the positive associations between media-based and interpersonal information-source acquisition and risk perception were weaker at higher levels of trust in authoritative information sources. This finding suggests that trust is not only a facilitator of information acceptance but may also operate as a filter that narrows the cues individuals use in risk appraisal. Overall, the study contributes to risk communication research by linking communication structure, risk appraisal, and cooperation-oriented protective behavior within a single empirical framework.

From a practical standpoint, this study offers the following guidance for governmental and public health agencies seeking to strengthen crisis communication and support cooperation-oriented protective behavior: First, build a pluralistic information ecology. Integrate authoritative outlets, mass media, and interpersonal networks, which may help broaden coverage and facilitate timely interaction, while avoiding dependence on any single source. Second, design targeted, actionable content. Tailor communication content to heterogeneous audience needs; provide specific, operable guidance to translate cognition into Behavior. Third, optimize narrative strategies. Balance the rational persuasiveness of data-based messaging with the emotional resonance of story-based formats to enhance identification and motivation to act. Fourth, strengthen media coordination. Promote coherent collaboration between traditional media and digital platforms to support consistent cross-platform communication and broaden message reach.

### 7.1 Limitations and future directions

This study has several limitations. First, the data are drawn from a cross-sectional survey: although geographically broad, the design is subject to self-report bias and potential social-desirability effects. Second, the causal chain between risk communication strategies and cooperation-oriented protective behavior has not been verified through longitudinal or experimental designs, making it difficult to rule out reverse causality and unobserved confounders. Third, key psychosocial variables, such as emotional responses, social norms, and cultural differences, were not incorporated into the model, and data collection was limited to a specific time frame, preventing the assessment of dynamic changes. In addition, because the dependent variable captures cooperation-oriented protective behavior rather than pure social-dilemma cooperation, future research should develop measures that separately assess self-protective motives, other-protective motives, and collective-action motives in public health contexts.

Future research can be extended in several directions: (1) Incorporate big-data tracking, eye-tracking experiments, or social media Behavior mining to improve the precision of measuring actual protective behaviors in real-world contexts. (2) Examine how emotional variables such as anxiety and subjective well-being, along with social norms and other psychosocial factors, shape the pathway from risk communication to cooperation-oriented protective behavior. (3) Conduct deeper comparisons of risk communication responses across different populations, such as older adults and university students, to inform more targeted and effective communication strategies. (4) The operationalization of the communication constructs also imposes measurement boundaries. This study captured recipient-side engagement with risk information through source acquisition, content attention, format preference, and channel reliance, rather than through alternative evaluative, cognitive, experimental, or behavioral measures. Future research could compare these operationalization with event-specific, behavioral, or experimental designs to examine whether the observed associations with risk perception and cooperation-oriented protective behavior remain consistent across different measurement approaches.

## Data Availability

The raw data supporting the conclusions of this article will be made available by the authors, without undue reservation.
